# Surge Dose^®^ Formulations of NSAIDs Provide for Ultra-Rapid and Consistent Drug Absorption in Both the Fasted and Fed State as Predicted by Physiologically Based Biopharmaceutics Modelling

**DOI:** 10.3390/pharmaceutics17060708

**Published:** 2025-05-28

**Authors:** Harri Dickinson, Zhixin Jiang, Paul A. Dickinson, Ian R. Wilding, Geraldine A. Elliott

**Affiliations:** 1 Seda Pharmaceutical Development Services, Oakfield Road Cheadle Royal Business Park, Cheadle, Stockport SK8 3GX, UK; harri.dickinson@sedapds.com (H.D.); zhixin.jiang@uk-koeln.de (Z.J.); paul.dickinson@sedapds.com (P.A.D.); 2 Ian Wilding Associates Limited, 10 Glebe Street, Nottingham NG9 1BZ, UK; ian@ianwildingassociates.com; 3 Imaginot Pty Ltd., Yeerongpilly Corporate Park, 44 Station Street, Yeerongpilly, Brisbane, QLD 4105, Australia

**Keywords:** PBBM (physiologically based biopharmaceutics modelling), diclofenac, ibuprofen, biorelevant dissolution, food effects, drug absorption, fast-disintegrating tablet

## Abstract

**Background/Objectives:** This paper describes the use of physiologically based biopharmaceutics modelling (PBBM) to predict the effect of food on diclofenac and ibuprofen absorption from ultra-rapid-release Surge Dose^®^ tablets. **Methods:** Fasted-state diclofenac pharmacokinetics (PK) were used with published IV data and biorelevant dissolution data for the diclofenac tablets to develop a mechanistic PBBM model which could be used to predict absorption. **Results:** The resultant model that best fitted the PK data showed that, in vivo, the ultra-rapid-release tablets behaved like a solution with a median time to peak plasma concentration (T_max_) of 20 min. Incorporating a well-established model for gastric emptying in the fed state, the fed T_max_ for these tablets was predicted to be 21 min, similar to that seen in fasted subjects. Use of a PBBM model to predict absorption of ibuprofen in the fasted and fed states again showed that ultra-rapid-release tablets produced fast and consistent absorption independent of the presence of food. Predicted mean T_max_ values were 31.8 and 35.4 min in the fasted and fed states, respectively. **Conclusions:** Therefore, even if Surge Dose^®^ formulations are taken after food, as frequently recommended for NSAIDs, the speed of absorption and subsequent onset of action should not be impacted.

## 1. Introduction

Physiologically based biopharmaceutics modelling (PBBM) is an evolving tool that is being increasingly used to inform and facilitate decision-making in the drug development process as well as gaining increasing acceptance by regulatory agencies as part of the product approval process [1]. This subset of physiologically based pharmacokinetic (PBPK) modelling integrates physicochemical drug properties, dissolution profiles and specific characteristics of the gastrointestinal (GI) tract to predict in vivo exposures and bioavailability in both fasted and fed states. PBPK models are constructed using a bottom-up approach, whereby the actual physiological characteristics are integrated to characterise pharmacokinetic (PK) behaviours in both the preclinical and clinical environment [2]. In contrast to empirical compartmental PK modelling methods, PBBM models enable the prediction of food effects [3] and the assessment of formulation performance [4] in in vivo drug absorption. Hence, such approaches provide a robust scientific basis for understanding the PK of drugs, for which there is increasing regulatory support.

This study used PBBM to facilitate a better understanding of the impact of food on the absorption and clinical outcomes of two widely used NSAIDs (nonsteroidal anti-inflammatory drugs), diclofenac and ibuprofen, when formulated as ultra-rapid-release tablets using the Surge Dose^®^ technology [5]. Both drugs are available in a variety of commercial products ideally taken after food to reduce the risk of GI side-effects which can also be minimised by using the lowest effective dose of these drugs [6]. Like many other drugs, diclofenac and ibuprofen demonstrate variable absorption in the fasted state [7,8], attributable to the impact of the migrating motor complex (MMC) on gastric emptying (GE), which is an important determinant of drug dissolution and absorption in the fasted state [9]. In the fed state, absorption is typically delayed and is more variable, being heavily influenced by the fed-state GE processes, especially for weak acids. Rapid, consistent absorption and onset of action for any analgesic are important, so any delays will potentially compromise clinical efficacy. Therefore, tablet formulations that provide fast and consistent drug absorption in both fasted and fed states, independent of the MMC and GE, are needed to provide optimal clinical outcomes.

Consistent with recent publications outlining the importance of the biopharmaceutics risk assessment [10,11] for optimised clinical drug product performance, the Surge Dose^®^ tablets have been designed to achieve ultra-rapid activated dissolution of a drug in co-administered liquid and gastric contents. This will then allow for the resultant solution to empty rapidly from the stomach independent of the MMC. This results in faster and more consistent absorption, as demonstrated previously for lornoxicam, diclofenac and paracetamol formulated as Surge Dose^®^ tablets in fasted PK studies in human volunteers [12]. More rapid absorption produces earlier and higher maximum plasma concentrations (C_max_), with earlier onset of analgesia as well as better overall and longer lasting analgesia. PK-PD modelling predicted faster and greater analgesia for Surge Dose^®^ paracetamol tablets within 30 min post-dosing compared with other commercial products [13]. Given the consumer need for fast and consistent onset of action from analgesic tablets, whether taken in the fasted state or after food, PBBM was identified as a computational modelling strategy to predict how Surge Dose^®^ formulations would be absorbed in the fed state without the need for lengthy and expensive biostudies.

## 2. Materials and Methods

### 2.1. Formulation Design and Biorelevant Dissolution Testing

Imaginot has developed its Surge Dose^®^ tablet formulation technology to provide ultra-rapid release in vivo, based on in vitro dissolution of at least 50% dissolution in 5 min under discriminating, non-sink in vitro conditions using 900 mL 0.0033 M hydrochloric acid at 30 rpm and 37 °C in USP dissolution apparatus II [5]. This test is used as a discriminating development tool to demonstrate the inherent ultra-rapid dissolution characteristics of these formulations that would be masked at higher stirring speeds. These conditions are biorelevant for this technology based on the demonstrated rapid and consistent drug absorption in fasted-human PK studies from tablets meeting this in vitro dissolution specification.

Surge Dose^®^ tablet formulations of diclofenac and ibuprofen were developed as described in [5], incorporating levels of pH-modulating agents combined with water-uptake agents and other conventional tablet excipients to maximise the drug dissolution rate under the biorelevant in vitro dissolution conditions. Film-coated Surge Dose^®^ diclofenac sodium 50 mg tablets were developed at full commercial manufacturing scale containing 600 mg sodium bicarbonate with 50 mg fumaric acid, 240 mg microcrystalline cellulose, 50 mg sodium starch glycolate and 10 mg magnesium stearate. They released 89% of the drug in 5 min under the biorelevant test conditions. PK evaluation in 21 fasted subjects showed rapid and consistent absorption with mean and median times to peak plasma concentration (T_max_) of 20 min (range 15–30 min). Prototype Surge Dose^®^ ibuprofen 200 mg tablets contained 600 mg sodium bicarbonate, 540 mg microcrystalline cellulose, 50 mg croscarmellose sodium and 10 mg magnesium stearate, releasing 88% ibuprofen in 5 min under the biorelevant test conditions.

### 2.2. Physiologically Based Biopharmaceutics Modelling (PBBM) Strategy

Ibuprofen and diclofenac are classified as BCS (Biopharmaceutics Classification System) Class IIa drugs based on their high permeability and low solubility under acidic conditions. Both are weak acids and, given the similarities of their physicochemical and PK properties, it was considered feasible to predict the absorption of ibuprofen Surge Dose^®^ tablets from modelling based on fasted PK data for diclofenac Surge Dose^®^ tablets. The PBBM approach allows for different systemic PK to be accounted for, and, therefore, if dissolution and absorption are sufficiently similar, based on the physicochemical properties of the compounds allowing for such an extrapolation, the utilised approach may also be suitable for other compounds with similar properties (e.g., NSAIDs and other weak acid drugs with high intestinal solubility and high permeability).

The aim was to build in silico PBBM models using the available fasted-human PK data for Surge Dose^®^ diclofenac tablets to define the fasted gastric mean transit time (MTT) or GE rate for such formulations. These models were then used to mechanistically describe the PK performance of diclofenac from these formulations in the fed state and then to describe PK performance in both the fasted and fed state for ibuprofen Surge Dose^®^ tablets.

**Figure 1:**
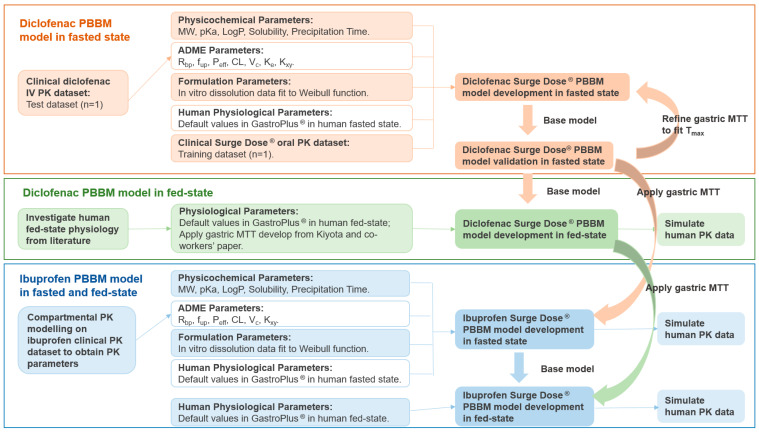
The workflow of the diclofenac and ibuprofen PBBM models’ development and simulation. MW: molecular weight; pKa: acid dissociation constant; LogP: octanol–water partition coefficient; R_bp_: blood-to-plasma ratio; f_up_: fraction unbound in plasma; P_eff_: effective permeability; CL: systemic clearance; V_c_: systemic volume of distribution of the central compartment; K_e_: elimination rate of constant; K_xy_: rate of constant from x to y; MTT: mean transit time; T_max_: time of maximum plasma concentration.

The workflow of the PBBM models’ development, verifications and simulations are presented in Figure 1 PBBM modelling was performed in GastroPlus^®^ v9.8.3 (SimulationPlus, Inc., Lancaster, CA, USA) using standard settings except for in the case of the gastric MTT, which was optimised to describe the unique properties of the ultra-rapid-release tablet formulations.

#### 2.2.1. Diclofenac Surge Dose^®^ PBBM in Fasted State

Diclofenac physicochemical properties were predicted using ADMET Predictor™ in GastroPlus^®^ with the results validated against reported values [14]. The human intravenous (IV) systemic PK parameters were derived from the current literature [15] and used to simulate a PK profile in a three-compartmental PK model for the Surge Dose^®^ diclofenac tablet. Equations and schematics for the three-compartmental PK model are shown in the supporting information (Equations (S3) and (S5)–(S9) and Figure S1c). A simulated PK profile was visualised and convoluted with the observed PK data, which were digitised from the same literature as the IV PK parameters. Validated systemic PK parameters were augmented by the default values predicted in ADMET Predictor™ as the initial input to the GastroPlus^®^ diclofenac PBBM model. It was assumed that 70% of the administered diclofenac is eliminated by the kidneys [16].

Diclofenac Surge Dose^®^ solubility and dissolution data were obtained from in vitro experiments. The dissolution data for diclofenac Surge Dose^®^ tablets were imported into GastroPlus^®^ as .dsd files and fitted to the Weibull function in GastroPlus^®^ using the one- or two-phase Weibull models described in the supporting information (Equations (S10) and (S11)).

The Advanced Compartmental Absorption and Transit (ACAT) model in GastroPlus^®^ with default human physiological settings in a fasted state (using the Opt LogD Model SA/V 6.1) was used to calculate the absorption scaling factors (ASFs).

The fitted diclofenac Surge Dose^®^ Weibull parameters and IV PK parameters were incorporated into the human PBBM model to provide a single dose simulation in the fasted state (Table 1). The model was subsequently refined by adjusting the gastric MTT to fit the T_max_ to the 50 mg observed diclofenac Surge Dose^®^ oral PK data. In order to test the boundaries of the model, partial parameter sensitivity analysis (PPSA) was undertaken around the gastric MTT, mean precipitation time (MPT) and in vitro dissolution.

#### 2.2.2. Diclofenac Surge Dose^®^ PK Prediction in Fed State

Two first-order phases of GE kinetics have been shown to occur in the fed state, with the first phase being due to the rapid emptying of ingested water unmixed with food along the lesser curvature of the stomach (colloquially known as the “Magenstraße”, or stomach road) and the slower second phase resulting from the emptying of food contents [18]. To model the kinetics of gastric secretion, a zero-order equation, a first-order equation and a combination of two first-order equations representing the early and late phases, respectively, were used. For the latter, a transition time of 1.5 h (i.e., when gastric secretion reaches a steady state) was applied.

It was assumed that the Magenstraße empties with a 5 min half-life, coinciding with the early phase of gastric secretion. The percentage contribution of the Magenstraße in the early phase of the drug GE kinetics varies depending on the formulation of the drug. Fast-dissolving diclofenac Surge Dose^®^ tablets achieved T_max_ in 20 min or less in the fasted condition, which is similar to that for an oral solution with a median T_max_ of about 15 min [19].

In the best-fit fed-state model used to evaluate the PK parameters of oral solutions [18], 75% of the drug content was emptied from the stomach in the initial 1.5 h (early phase) until the gastric secretion reached steady state, and the remaining 25% of the drug content started to empty after 1.5 h (late phase). The equations are shown in the supporting information (Equations (S12)–(S15)) for the early phase and late phase, respectively. The correlation between the GE constant rate (*K_GE (fed)_*) and the gastric *MTT* (in units of h) as the GastroPlus^®^ input was investigated, as shown in Supplementary S3, and was then applied to the different phases (early and late) in the PBBM simulation.

Therefore, a single 50 mg dose of Surge Dose^®^ diclofenac, comprising 37.5 mg (75% of the total dose) dosed at the 0 h time point and 12.5 mg (25% of the total dose) dosed at the 1.5 h time point, was simulated in the fed state by incorporating the fasted PBBM model developed previously and the fed physiology described by Kiyota’s model. The two phases of the “single dose” in GastroPlus^®^ can be performed in two ways. The first method involves separate simulations corresponding to the early and late phases, assuming there is no interaction between the first 75% and the remaining 25% dose (i.e., there is no solubility limitation). This means they can be treated as two distinct entities and the individual PK profiles can then be summed to produce a combined PK profile. The second method employs single simulations within a multiple-dose dosing file, applying different physiologies for gastric emptying to the first dose (i.e., 75%) and second dose (i.e., 25%). This latter approach is more appropriate if there is likely to be an interaction between the two doses in the GI tract such as a solubility limitation. The simulation results for both methods were found to be consistent for the single-subject simulation, confirming that there was no interaction between the two fractions of the dose. This was expected based on the rapid dissolution and high intestinal solubility of NSAIDs. However, as virtual trials cannot be performed in GastroPlus^®^ using the multiple dosing file approach, the combined “separate dose” approach was applied, as described later for virtual trials.

#### 2.2.3. Ibuprofen Surge Dose^®^ PK Prediction in Fasted and Fed States

Ibuprofen is administered as a racemic mixture of R and S enantiomers, with S-ibuprofen being largely responsible for its pharmacologic activity. A previous study [20] reported plasma concentrations for each enantiomer; however, these were summed for PK analysis. This approach was considered appropriate as, generally, ibuprofen PK studies do not employ enantiomeric selective assays. In R^®^, 2.5 mg/kg R/S-enantiomers ibuprofen IV PK data [20] were digitised, cleaned and visualised [21]. One-compartment and two-compartment PK models were subsequently fitted to the digitised total concentration of R/S enantiomers (racemic) versus the time profile. The mathematical equations that govern the one-compartment PK model are defined in the supporting information (Equations (S1), (S6) and (S9)), and the two-compartment PK model is defined in the supporting information (Equations (S2), (S4), (S6), (S7) and (S9)). Non-compartment analysis (NCA) was conducted by using the “PKNCA” package in R^®^ [22] to calculate starting values of the PK parameters.

Biorelevant ibuprofen Surge Dose^®^ dissolution data were loaded as .dsd files and fitted to the Weibull function™ in GastroPlus^®^ using the one- or two-phase model described in the supporting information (Equations (S10) and (S11)). The physicochemical properties of ibuprofen were determined from the literature [23] and supplemented by predictions from ADMET Predictor™.

The ibuprofen-specific physicochemical parameters, the dissolution Weibull model of ibuprofen Surge Dose^®^ tablets, the gastric MTT fitted from the diclofenac fasted PBBM model (based on human PK data) and the simulated systemic PK parameters were used in GastroPlus^®^ to predict the performance of 400 mg ibuprofen Surge Dose^®^ tablets within a single fasted subject. The fitted gastric MTT from the diclofenac Surge Dose^®^ fasted PBBM model developed previously was applied to the ibuprofen simulations. The input parameters in GastroPlus^®^ for conducting PBBM are shown in Table 2. The simulation time was set to 4 h to ensure the Gastroplus^®^ assigned sample times had sufficient resolution for assessing the C_max_ and T_max_.

The fasted-state PK of a 400 mg ibuprofen solution were predicted using the model and compared with those of the Surge Dose^®^ tablets under the same dietary conditions to assess the impact of the Surge Dose^®^ formulation on the speed of drug absorption. The simulation time was once again set to 4 h.

In the next step, a virtual trial in which virtual subjects are created to reflect the clinical variability observed in key parameters affecting drug disposition, including gastrointestinal physiology, was performed utilising 24 subjects based on the ibuprofen single-subject (mean) simulation in the fasted state. Virtual trials allow intra-subject variability in PK, and any interaction with formulation performance to be understood and visualised in the same way as when running a clinical study. Such an approach can provide for greater insights than performing a ‘mean’ simulation only with standard physiological settings. Default variabilities were applied to all parameters excluding the Weibull fitted parameters and dose. The simulation time was set to 6 h and 12 h. Results from 12 h of simulation were compared with published ibuprofen lysine PK data [24,25].

A virtual trial containing 24 subjects administered with 400 mg ibuprofen as Surge Dose^®^ tablets in a fed state was simulated by using the PBBM model built in the fasted state and applying the Kiyota’s fed-state physiology developed above for diclofenac. Specifically, 300 mg ibuprofen (75% of the total dose) was administered at the 0 time point with early-phase physiology as the first simulation, and 100 mg ibuprofen (25% of the total dose) was administered at 1.5 h with late-phase physiology as the second simulation. Applying a cross-over design ensured that the same subjects/study population was used for both phases (if a single-arm virtual trial was run twice, then the two trials would be made up of different subjects as a new population is generated each time based on the default variabilities applied to each variable/parameter). It was found that the multiple dosing setting approach could not be applied in the virtual trial setting in GastroPlus^®^ as it was not able to deal with the two GE rates/physiologies required and rather applied the rapid GE physiology to both dose events. The total predicted concentrations were calculated by adding the values of the two profiles together. The simulation time was set to 12 h. This approach was considered acceptable as a physiological interaction between the two proportions of the dose was not expected since there is no solubility limitation if both proportions of the dose partially overlap in any part of the GI tract. While the modelling approach can be verified for the fasted situation using published clinical data, it is recognised that this is not the case in the fed state, especially for the Surge Dose^®^ tablets. However, the gastric emptying model applied is based on the analysis of clinical data for solutions dosed in the fed state [18]. Therefore, the analysis shared in this paper strongly indicates that Surge Dose^®^ tablets produce solution-like PK in the fed state.

## 3. Results

### 3.1. Diclofenac Surge Dose^®^ PBBM

#### 3.1.1. Diclofenac Surge Dose^®^ PBBM Fasted State

**Figure 2:**
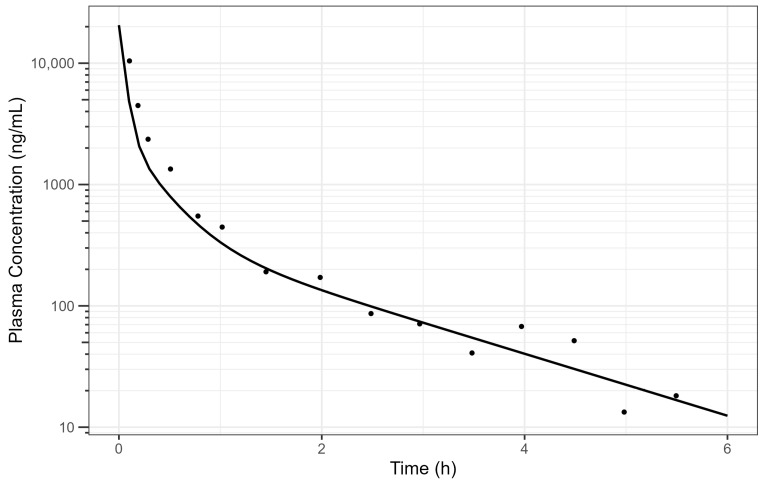
Simulated human diclofenac IV data using obtained PK parameters [15] where the line is the simulated PK profile, and the points are the raw data. The plot is on a log–linear scale.

The digitised and cleaned 50 mg diclofenac IV PK data with the simulated PK profile using fitted parameters are provided in Figure 2 and the selected PK parameters provide a good description of the data.

**Figure 3:**
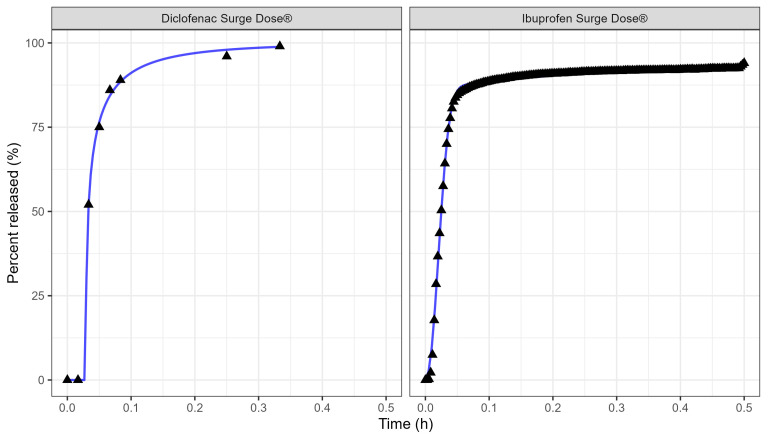
Weibull model fits of diclofenac Surge Dose^®^ (**left** plot) and ibuprofen Surge Dose^®^ (**right** plot) tablets in GastroPlus^®^ where the solid lines are the fitted dissolution profiles, and the triangular points are the raw data.

Table 3 and Figure 3 display the Weibull parameters (as applied to the PBBM prediction) for the Surge Dose^®^ diclofenac tablet and Surge Dose^®^ ibuprofen tablet dissolution curves.

**Figure 4:**
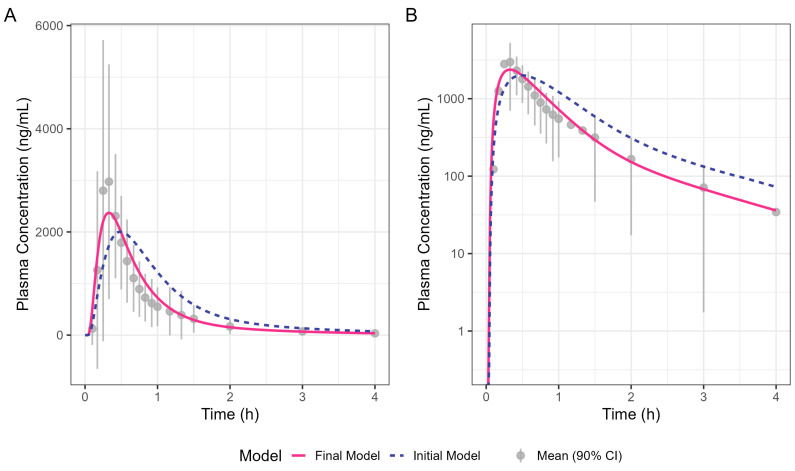
Oral diclofenac Surge Dose^®^ tablet human PK prediction. The solid line was predicted by the final gastric MTT optimised PBBM model, the broken line was predicted by the initial PBBM model with default settings for the gastric MTT and points with an error bar are the raw mean data with a 90% confidence interval (CI). (**A**) Plot is on a linear–linear scale. (**B**) Plot is on a log–linear scale.

Figure 4 shows the simulated human Surge Dose^®^ diclofenac concentration–time profiles from the initial and optimised final PBBM models. The initial model was refined by increasing the total clearance by 30% and adjusting the gastric MTT to capture the observed T_max_. Clearance was increased to ensure a good fit to the terminal phase, which is not governed by absorption kinetics. An adjustment of 30% was within the realms of what is feasible, allowing for intra-subject differences between the Surge Dose^®^ and IV data, which were from two different clinical studies. A gastric MTT of 0.1 h is recommended by SimulationsPlus™ in GastroPlus^®^ when modelling the PK performance of a solution rather than 0.25 h for a tablet. The simulation achieved by refining the gastric MTT is shown in Figure 4 as a solid line which better fits the observed data compared with the broken line for the original model. The close fit of the 19.8 min T_max_ for the final model with the solution gastric MTT provides evidence that Surge Dose^®^ diclofenac tablets act as a solution in vivo due to their rapid disintegration and dissolution characteristics.

**Figure 5:**
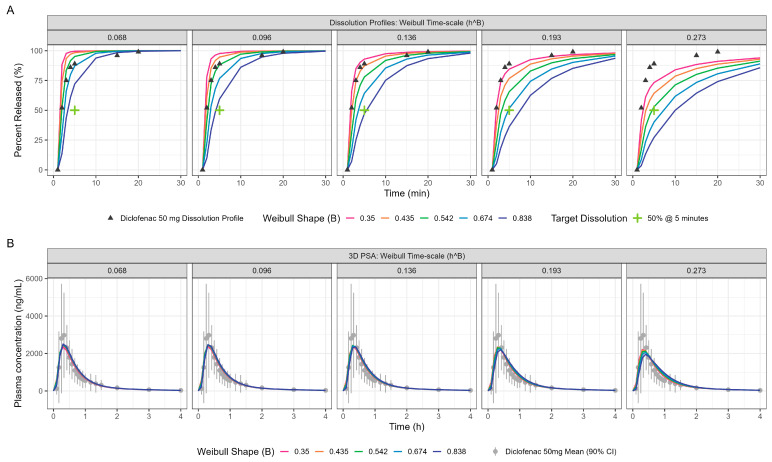
PPSA showing the impact of in vitro dissolution changes (panel (**A**)) on the resulting PK (panel (**B**)) for oral diclofenac Surge Dose^®^ tablets in the fasted state. The Surge Dose^®^ prototypes were designed to have an in vitro dissolution of at least 50% dissolution in 5 min under biorelevant conditions. This minimum dissolution is marked with a green cross in panel (**A**).

PPSA assessment of the diclofenac fasted model yielded further insights on the interplay of in vitro dissolution and the gastric emptying rate for these ultra-rapid-release Surge Dose^®^ tablets. The impact of changing in vitro dissolution on PK, especially the Cmax and Tmax, is presented in Figure 5 and Table 4. Sensitivity analyses around the MTT and MPT are provided in the supporting information (Figures S3 and S4, respectively).

#### 3.1.2. Diclofenac Surge Dose^®^ Tablet PBBM in Fed State

**Figure 6:**
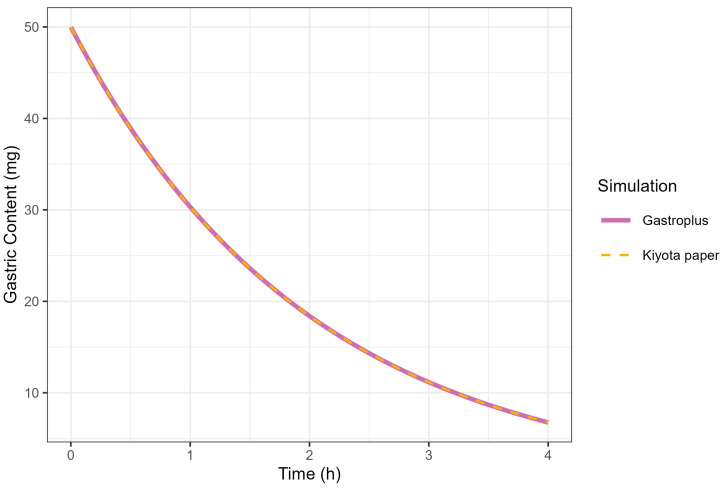
Simulation of gastric contents at 50 mg dose from GastroPlus^®^ (solid line) and fed-state physiology derived from Kiyota’s paper (dashed line).

The correlation between the K_GE_ and MTT was investigated under conditions where the MTT was fixed at 2 h. A K_GE_ value of 0.5 h successfully reproduced the GE profile simulated by GastroPlus^®^ (MTT = 2 h), consistent with the mathematical relationship defined in Equation (1). The complete superimposition of GE curves generated by GastroPlus^®^ (using MTT = 2 h) and Kiyota’s model [18] (using KGE = 0.5 h) provided empirical validation of the functional equivalence between these parameters, thereby confirming the theoretical correlation (Figure 6).

(1) $$K_{GE(fed)}=1/MTT,$$

**Figure 7:**
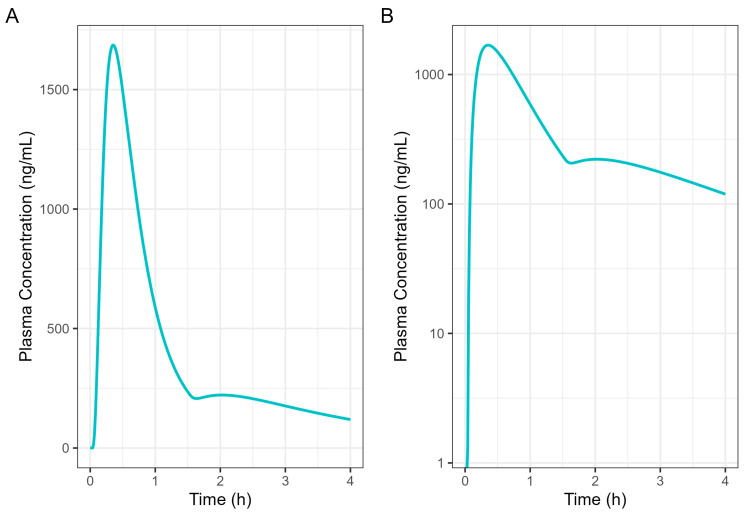
Diclofenac human 50 mg Surge Dose^®^ tablet PK prediction in the fed state. (**A**) Plot is on a linear–linear scale. (**B**) Plot is on a log–linear scale.

Figure 7 presents a single-subject simulation of absorption from a 50 mg Surge Dose^®^ diclofenac tablet in the fed state based on the results of the GE investigation. In the simulation, 37.5 mg (75%) of the diclofenac dose was administered at the 0 time point with a gastric MTT of 0.14 h as the early-phase emptying, followed by 12.5 mg (25%) of the diclofenac dose at 1.5 h with a gastric MTT of 2.21 h as the late-phase process.

#### 3.1.3. Comparison of Fasted and Fed Results

Table 5 provides a comparison of the single simulation output for 50 mg Surge Dose^®^ diclofenac tablets in fasted and fed states. While food decreases C_max_ (µg/mL) from 2.97 to 1.69, there is a negligible increase in T_max_ from around 19.8 min to 21.0 min from the fasted to the fed state. AUC_(0–4 h)_ values are similar (<5% difference) for fasted and fed states, indicating that overall exposure is not impacted by food. Previous researchers [19] compared a solution of diclofenac formulated with potassium bicarbonate with an immediate-release tablet in fasted and fed states. The results demonstrated a minor impact of food on T_max_ for the diclofenac solution, with a difference of approximately 5 min between the fasted and fed states. The findings indicated that Surge Dose^®^ diclofenac tablet formulations exhibited a similar pattern of behaviour to that observed with a solution, i.e., faster and more consistent absorption in the presence of food.

### 3.2. Ibuprofen Surge Dose^®^ PBBM

#### Ibuprofen Surge Dose^®^ PBBM in Fasted State

The fitted model parameters for the ibuprofen IV data are provided in Table 6. Model fits can be seen in the supporting information (Figure S2).

**Figure 8:**
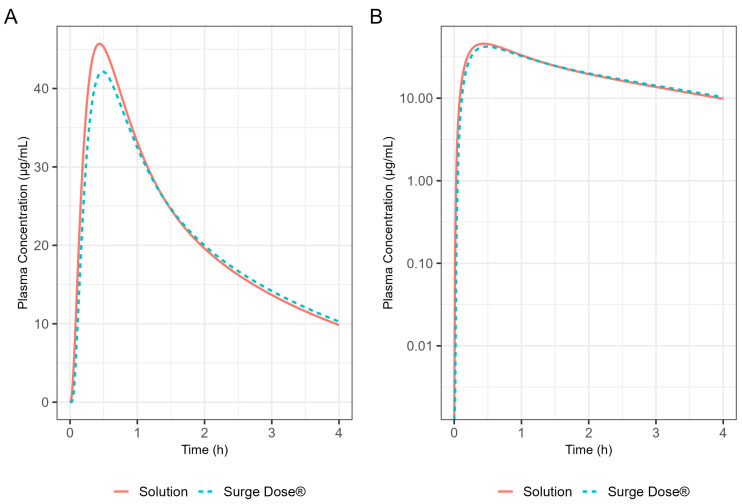
Simulated 400 mg ibuprofen PK from administration of a Surge Dose^®^ tablet (broken line) or an aqueous solution (solid line) in the fasted state for a single standard subject (i.e., standard GastroPlus^®^ setting). (**A**) Plot is on a linear–linear scale. (**B**) Plot is on a log–linear scale.

**Figure 9:**
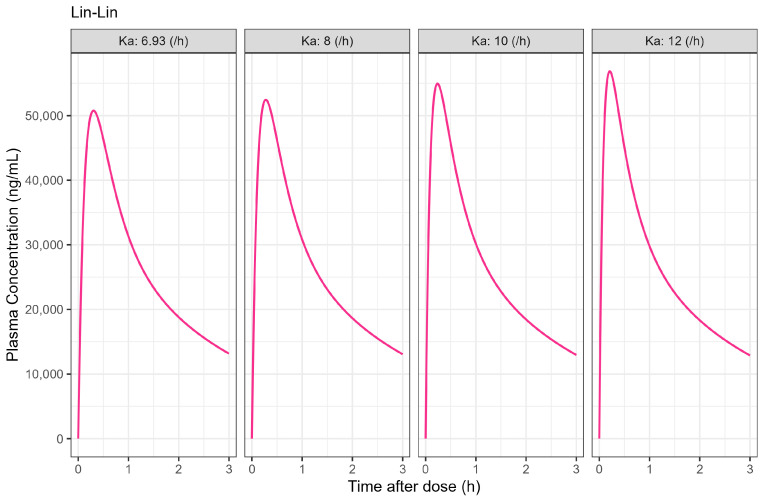
Simulated 400 mg single oral dose of ibuprofen PK profiles with assumed different absorption rate constant (unit: /h) in two-compartmental PK model. PK parameters were derived from Table 6 and estimated in PK compartmental models and oral bioavailability was set to 1.

The Weibull model fit of the in vitro ibuprofen Surge Dose^®^ tablet dissolution profile is provided in Table 3 and Figure 3. The 0.1 h gastric MTT from the diclofenac model and the fitted Weibull model were applied to the ibuprofen oral Surge Dose^®^ tablet fasted-state PBBM simulation. The predicted single-subject PK for the Surge Dose^®^ tablet and solution are shown in Figure 8 and Table 7. The Surge Dose^®^ tablet formulation shows an ultra-rapid absorption with a T_max_ of 28.8 min, only 2.4 min later than that of the solution. Figure 9 illustrates a simple compartmental PK model simulation using the two-compartment PK model for ibuprofen and assuming a first-order input/absorption rate. These simulations demonstrate that, even with a very rapid absorption rate, the shortest T_max_ it is possible to achieve is 17.4 min (absorption half-life <3.5 min (ka = 12/h)). This highlights an often forgotten observation: that T_max_ is not solely dependent on absorption rate but also the systemic PK parameters. This means that formulations with the same rapid-release characteristics but containing different drugs can present with different T_max_ values due to differences in the systemic clearance of the individual drugs.

### 3.3. Population Simulations for Surge Dose^®^ Ibuprofen in Fasted and Fed Subjects

**Figure 10:**
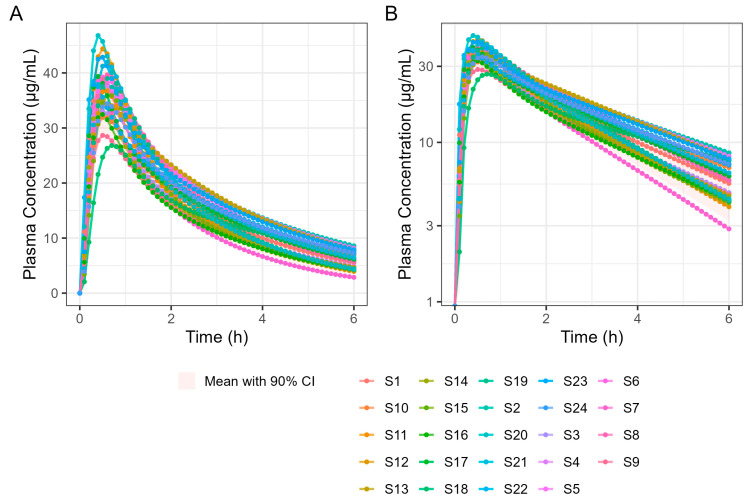
Population simulation of 400 mg Surge Dose^®^ ibuprofen tablets in the fasted state. (**A**) Plot is on a linear–linear scale. (**B**) Plot is on a log–linear scale.

A 4 h simulation run was undertaken to allow a detailed examination of T_max_. Subsequent simulations were performed for 6 and 12 h in a virtual trial simulation with 24 fasted subjects and the results are summarised in Table 8. It should be noted that the results differ slightly because GastroPlus^®^ automatically assigns sample times based on simulation length, which means the two simulations are based on different sample times. Figure 10 shows the results of the 6 h population fasted PK simulation on 24 subjects.

**Figure 11:**
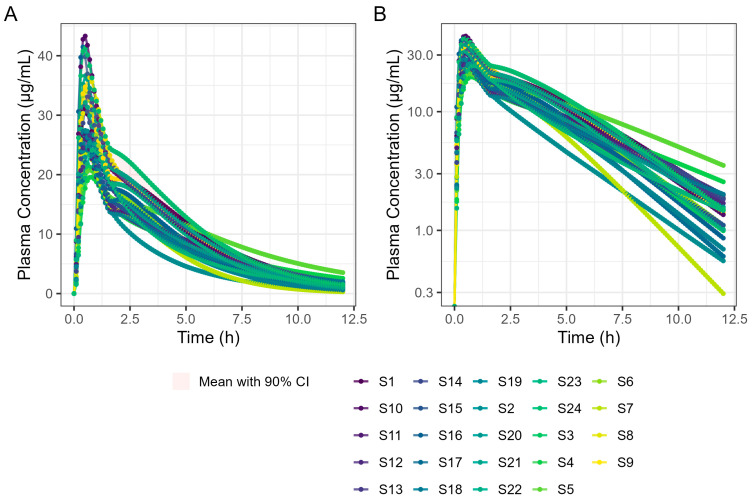
Population simulation of 400 mg Surge Dose^®^ ibuprofen tablets in the fed state. (**A**) Plot is on a linear–linear scale. (**B**) Plot is on a log–linear scale.

The virtual trial of 400 mg Surge Dose^®^ tablet PK prediction in the fed state for the 24 human subjects is shown in Figure 11. Simulated PK parameters are shown in Table 9. The T_max_ statistics obtained from fasted and fed simulations are summarised in Table 10.

These results predict that dosing Surge Dose^®^ ibuprofen tablets in the fed state will result in a similar median T_max_ to the fasted state: 30 min. This contrasts with the significant increase in T_max_ and associated decrease in C_max_ that are common in the fed state for fast-acting compounds and have a consequential impact on the time and extent of the analgesic effect [26]. Ibuprofen Surge Dose^®^ AUC_0–12_ predictions were similar in fasted and fed states at around 116 µg.h/mL, suggesting a similar extent of absorption from Surge Dose^®^ ibuprofen tablets whether dosed with or without food.

## 4. Discussion

This paper shows how a PBBM approach can be used to provide greater insights into the in vivo performance of new tablet formulations, particularly in the fed state, based on biorelevant in vitro dissolution characteristics and fasted PK data. This can significantly reduce development time and costs, minimising extensive PK studies in humans.

The results demonstrate that diclofenac and ibuprofen delivered as ultra-rapid-release Surge Dose^®^ tablets are absorbed like liquids in the fasted state, which allows similarly rapid absorption in the fed state. This is an important finding for drugs taken for acute indications such as pain, particularly NSAIDs, which are frequently taken after a meal to minimise the potential for GI side-effects and the risk of subsequent delayed and increased variability of absorption dependent on GE. Since rapid absorption of ibuprofen leads to an earlier and higher C_max_, associated with earlier onset of action combined with enhanced overall and prolonged analgesia [26], the ideal analgesic formulation needs to provide fast and consistent onset of action whether the dose is taken with or without food.

Ultra-rapid-release Surge Dose^®^ tablet formulations have been developed to release at least 50% of the drug under highly discriminating and biorelevant, non-sink in vitro test conditions within the first 5 min. These dissolution conditions use 900 mL of 0.0033 M hydrochloric acid at 37 °C with low stirring speeds as a development tool to achieve maximal dissolution under minimal agitation. Tablets of lornoxicam, diclofenac and paracetamol that meet this release specification have been shown to achieve significantly faster and more consistent absorption in fasted subjects compared with conventional solid oral dosage forms, which typically achieve less than 5% dissolution after 20 min under these biorelevant dissolution conditions. The employment of PBBM has allowed prediction of the absorption kinetics from these ultra-rapid-release tablets in the fed state, avoiding the need for expensive and time-consuming studies. Furthermore, it has enabled the prediction of the absorption of other drugs from such tablets in both fasted and fed states based on in vitro dissolution data collected under these biorelevant test conditions.

Fitting fasted diclofenac PK data to an established PBBM model has provided valuable insights into the absorption kinetics for these ultra-rapid-release tablets in the fasted state. An excellent fit for the human PK data with a T_max_ value of 20 min was achieved using GastroPlus^®^ standard settings by reducing the gastric mean transit time to 0.1 h. This finding provides compelling evidence that the rapid disintegration and dissolution of the drug from the Surge Dose^®^ tablet in vivo provide absorption kinetics similar to an aqueous solution where disintegration and dissolution are not rate limiting for absorption. Rapid dissolution of the drug in the co-administered water and gastric contents provides an optimal tablet formulation that behaves like a solution, facilitating earlier and more consistent onset of analgesia.

PPSA assessment of the fasted diclofenac model has shown that the gastric MTT of 0.1 h for the Surge Dose^®^ tablet formulations is actually a conservative value and could in fact be even faster to match the observed rate of absorption in the clinic. However, values faster than 0.1 h would be more rapid than the default value in GastroPlus^®^ for solutions, which suggests further characterisation of the gastric emptying of liquids and the subsequent impact on PK may be valuable, especially for highly permeable drugs with a short half-life (where C_max_/T_max_ is very sensitive to gastric emptying). Additional PPSA around the MPT has confirmed that, because of the unique performance of Surge Dose^®^ formulations, precipitation does not occur in the stomach and is also not relevant for a highly permeable weak acid in the small intestine. As a consequence, there were no scientific barriers to increasing the MPT from the GastroPlus^®^ default of 900 s.

PPSA was also used to investigate the impact of changes in dissolution rate outside the target of >50% in 5 min used in development of the Surge Dose^®^ formulations. The results presented in Figure 5 and Table 4 clearly demonstrate that the slowing of tablet dissolution for the utilised mean gastric MTT had a consequential impact on C_max_ with a subtle delaying of T_max_. These findings will be extended in due course to the establishment of safe space dissolution for the Surge Dose^®^ products.

By incorporating systemic PK parameters for ibuprofen with biorelevant dissolution data on a prototype Surge Dose^®^ tablet into the PBBM model that fitted the fasted diclofenac PK data, rapid and consistent absorption for the ultra-rapid-release ibuprofen tablet, again comparable to a solution, was predicted in the fasted state. A single-subject simulation for an ibuprofen solution and a Surge Dose^®^ tablet predicted T_max_ values of 26.4 min and 28.8 min, respectively. In the virtual trial of 24 subjects, T_max_ values ranged from 18.0 to 42.0 min for Surge Dose^®^ ibuprofen with mean and median T_max_ values of 31.8 min and 30.0 min, respectively. Similar mean and median T_max_ values are indicative of a normal distribution of absorption profiles with few slow or multiple peak profiles, attributable to GE being rate limiting for absorption. This compares with the shortest published mean T_max_ value of 32.52 min for ibuprofen lysine tablets [24].

Of interest were the different T_max_ values for diclofenac and ibuprofen, even for a solution of the drug, with T_max_ for ibuprofen being longer at 26–30 min than for diclofenac (around 20 min). This reflects that, although T_max_ and C_max_ are generally used as indicators of absorption rate, they are also dependent on the systemic PK for each drug. When comparing different drugs with different systemic PK characteristics, the post-absorption processes may have a greater influence on T_max_ than the dissolution characteristics of the formulation. In this case, the PBBM reveals that even the fastest dissolving ibuprofen tablet will not have as short a T_max_ as the fastest-dissolving diclofenac tablet.

In the fed state, rapid dissolution of the drug from the Surge Dose^®^ tablets in the co-administered water takes advantage of the early-phase gastric Magenstraße, producing a solution such that 75% of the dose will reach the small intestine for absorption in the first 90 min post-dosing. The remaining 25% will mix with stomach contents and reach the small intestine because of late-phase emptying. The fed GastroPlus^®^ PBBM diclofenac model suggests that there would be little impact of food on the absorption of this drug from an ultra-rapid-release tablet. The predicted fed-state T_max_ was 21.0 min compared with 19.8 min in the fasted state. This has important clinical implications as, when such a formulation is taken with food, it will avoid the delayed and variable absorption and associated variable onset of action observed with conventional tablets. A significant predicted benefit to the patient with this Surge Dose^®^ tablet formulation that behaves like a solution is that food will have little effect on absorption and onset of action, which is an important clinical benefit for such formulations.

Application of the diclofenac PBBM to ibuprofen predicted little effect of food on T_max_ values for Surge Dose^®^ ibuprofen tablets, consistent with the ultra-rapid dissolution of the drug in vivo where the tablet behaves like a solution. While the median T_max_ value of 30 min was the same for fasted and fed states, the predicted mean fed T_max_ value was only slightly longer, 35.4 min compared with 31.8 min for fasted subjects. Predicted mean AUC_(0–12 h)_ values were similar for fasted and fed subjects, confirming that the presence of food did not reduce the overall exposure to the drug. In contrast, absorption from conventional ibuprofen acid tablet was delayed from a T_max_ of 53 min to a T_max_ of 93 min by food, and even a fast-acting ibuprofen lysine tablet showed a significant food delay, from a T_max_ of 33.0 min in the fasted state to a T_max_ of 70.8 min after food [27].

Of particular note is that the predicted fasted and fed T_max_ values for Surge Dose^®^ ibuprofen tablets are faster and more consistent than the T_max_ values reported in the literature for conventional tablet formulations (Table 11).

Reported mean or median T_max_ values for ibuprofen lysine tablets in the fasted state are in the range of 30–45 min, typically with high variability, as demonstrated by CVs of 30–40% and ranges of 25 to 90 min. The simulated Surge Dose^®^ ibuprofen T_max_ values in the fasted state predicted more consistent absorption with similar mean and median values for T_max_ of 31 and 30 min, respectively, but with significantly lower variability (17% CV) and a narrow range of 18 to 42 min. Published T_max_ data on ibuprofen lysine tablets in the fed state show a significant delay in absorption, with median values of 70 to 90 min and an extended range from 30 to 180 min. In contrast, the fed simulated data for Surge Dose^®^ ibuprofen predict faster and more consistent absorption, with mean and median T_max_ values around 30 min with a narrower range of 24 to 54 min.

Similar simulated Surge Dose^®^ ibuprofen T_max_ data for both fasted and fed states suggest that such formulations will offer rapid absorption and consistent clinical outcomes irrespective of whether the dose is taken on an empty stomach or not. This rapid in vivo dissolution and absorption also has clinical benefits in terms of reducing the potential for GI damage with ibuprofen and other NSAIDs, improving their GI tolerability. Rapid dissolution in the stomach converts the drug to the ionised species, which is less absorbed by the gastric mucosa and avoids direct local damage from undissolved drug in contact with the mucosa. The short gastric residence time of the dissolved drug predicted by the model for these rapidly disintegrating tablets has the potential to also reduce the time over which gastric damage can occur.

## 5. Conclusions

This work demonstrates that PBBM can be an effective tool to facilitate the understanding of food effects on the absorption of drugs such as diclofenac and ibuprofen from ultra-rapid-release tablets with the potential to reduce development time and costs by reducing the need for fed clinical studies. PBBM using fasted PK data for a Surge Dose^®^ tablet of diclofenac that meets a biorelevant in vitro release specification of greater than 50% dissolution in 5 min indicated that such a tablet behaves as a solution in vivo. Applying established modelling for gastric emptying in the fed state allows prediction of fed-state PK and suggests little impact of food on T_max_. Therefore, even if Surge Dose^®^ formulations are taken after food, as frequently recommended for NSAIDs, the speed of absorption and subsequent onset of action should not be impacted.

## Figures and Tables

**Table 1:** Initial settings of the diclofenac PBBM model.

Parameter	Initial Value	Reference
MW (g/mol)	296.15	ADMET Predictor™
LogP	4.51	ADMET Predictor™
pKa	Acid 4.15	ADMET Predictor™
P_eff_ (×10^−4^ cm/s)	6.4	ADMET Predictor™
Body weight	63.4	[15]
CL (L/h)	21.02	[15]
V_c_ (L/kg)	0.039	[15]
K_12_ (/h)	5.5	[15]
K_21_ (/h)	5.2	[15]
K_13_ (/h)	2.6	[15]
K_31_ (/h)	0.8	[15]
K_e_ (/h)	8.5	[15]
Solubility (mg/mL)	1.618 at pH 6.8	Imaginot in-house data
Solubility scaling factor	643.82	ADMET Predictor™
Mean precipitation time (seconds)	30,000	Set nominally high as no precipitation was assumed
Physiology	Human-Physiological-Fasted	Default in GastroPlus^®^
ASF model	Opt logD Model SA/V 6.1	Default in GastroPlus^®^
Plasma protein binding (f_up_) %	0.05	[17]
Blood-to-plasma ratio	1	Assumed

**Table 2:** Initial settings of the ibuprofen PBBM model.

Parameter	Initial Value	Reference
MW (g/mol)	206.29	ADMET Predictor™
LogP	3.23	[23]
pKa	Acid 4.5	[23]
P_eff_ (×10^−4^ cm/s)	17	[23]
Body weight	71.8	[20]
PK parameters	Fitted in the previous step	
Solubility (mg/mL)	0.043 at pH 3	[23]
Solubility scaling factor	79	[23]
Mean precipitation time (seconds)	30,000	Set nominally high as no precipitation was assumed
Physiology	Human-Physiological-Fasted	Default in GastroPlus^®^ Gastric MTT fitted from diclofenac Surge Dose^®^ fasted PBBM model
ASF model	Opt logD Model SA/V 6.1	Default in GastroPlus^®^
Plasma protein binding (f_up_) %	0.01	ADMET Predictor™
Blood-to-plasma ratio	0.7	ADMET Predictor™

**Table 3:** Weibull parameters for diclofenac and ibuprofen Surge Dose^®^ tablet dissolution curve fits.

Phase	Parameter	Unit	Fitted Value	
			Diclofenac Surge Dose^®^	Ibuprofen Surge Dose^®^
	t (Time lag)	h	0.02923	0
	Max (Total released)	%	100	100
1	f	fraction	1	0.846
	A (Time scale)	h^b	0.13638	3 × 10^−4^
	b (Shape)	-	0.41876	2.24
2	f	fraction	-	0.153
	A (Time scale)	h^b	-	0.808
	b (Shape)	-	-	0.621

**Table 4:** PPSA for the Weibull parameters describing the impact of in vitro dissolution changes on C_max_ and T_max_ for the Diclofenac Surge Dose^®^ tablet in the fasted state. The values in green are the most similar to the actual measured dissolution profile.

Weibull Shape (B)	Weibull Time Scale (h^B)	Dissolution ≥50% in 5 min	C_max_ (ng/mL)	T_max_ (h)
0.35	0.068	Y	2344	0.3
0.435	0.068	Y	2436	0.3
0.542	0.068	Y	2466	0.3
0.674	0.068	Y	2481	0.3
0.838	0.068	Y	2502	0.3
0.35	0.096	Y	2378	0.3
0.435	0.096	Y	2445	0.3
0.542	0.096	Y	2466	0.3
0.674	0.096	Y	2471	0.3
0.838	0.096	Y	2482	0.352
0.35	0.136	Y	2389	0.3
0.435	0.136	Y	2428	0.3
0.542	0.136	Y	2426	0.3
0.674	0.136	Y	2422	0.352
0.838	0.136	N	2391	0.352
0.35	0.193	Y	2343	0.3
0.435	0.193	Y	2351	0.352
0.542	0.193	Y	2337	0.352
0.674	0.193	Y	2291	0.352
0.838	0.193	N	2196	0.352
0.35	0.273	Y	2222	0.352
0.435	0.273	Y	2207	0.352
0.542	0.273	Y	2154	0.352
0.674	0.273	N	2060	0.352
0.838	0.273	N	1951	0.4

**Table 5:** Summary statistics for 50 mg Surge Dose^®^ diclofenac tablet PBBM single simulation in the fasted and fed states.

Prandial State	Parameter	Unit	Value
Fasted	C_max_	µg/mL	2.97
	T_max_	minutes	19.8
	AUC_(0–4 h)_	µg.h/mL	1.90
Fed	C_max_	µg/mL	1.69
	T_max_	minutes	21.0
	AUC_(0–4 h)_	µg.h/mL	1.99

**Table 6:** Ibuprofen two-compartment human PK model parameters estimate with 95% CI and relevant GastroPlus^®^ input.

Parameter	Unit	Estimated Value (95% CI)	GastroPlus^®^ Input
Clearance	L/h/kg	0.0466 (0.0443, 0.049)	0.042 for kidney clearance 0.0047 for other clearance
V	L/kg	0.0742 (0.085, 0.0798)	0.0742
K_12_	/h	0.813 (0.62, 1.01)	0.813
K_21_	/h	1.23 (1.01, 1.45)	1.23

**Table 7:** Summary statistics for 400 mg ibuprofen PBBM single simulation in solution and Surge Dose^®^ tablet in the fasted state.

Prandial State	Dosage Form	F_a_	C_max_ (µg/mL)	T_max_ (Minutes)	AUC_(0–4 h)_ (µg.h/mL)
Fasted	Solution	100	45.7	26.4	119
	Surge Dose^®^	99	42.2	28.8	119

**Table 8:** Summary statistics for ibuprofen 400 mg Surge Dose^®^ tablet PBBM population simulation in the fasted state.

Prandial State	Simulation Time	Parameter	Unit	Arithmetic Mean	CV (%)	Median	Minimum	Maximum
Fasted	6 h	C_max_	µg/mL	36.97	12.58	37.31	26.82	46.79
		T_max_	minutes	31.2	17.07	30.0	18.0	42.0
		AUC_(0–6 h)_	µg.h/mL	117.7	15.41	120.2	84.05	149.5
	12 h	C_max_	µg/mL	36.95	12.67	37.25	26.62	46.66
		T_max_	minutes	31.8	18.05	30.0	18.0	42.0
		AUC_(0–6 h)_	µg.h/mL	116.8	15.03	116.2	85.0	150.30

**Table 9:** Summary statistics for ibuprofen 400 mg Surge Dose^®^ tablet PBBM population simulation in the fed state.

Prandial State	Simulation Time	Parameter	Unit	Arithmetic Mean	CV (%)
Fed	12 h	C_max_	µg/mL	29.9	22.3
		T_max_	minutes	35.4	23.3
		AUC_(0–12 h)_	µg.h/mL	116	18.9

**Table 10:** Summary statistics for ibuprofen 400 mg Surge Dose^®^ tablet PBBM population simulation in the fed state compared with the fasted state.

Prandial State	Simulation Time	Parameter	Unit	Arithmetic Mean	CV (%)	Median	Minimum	Maximum
Fasted	12 h	T_max_	minutes	31.8	17.1	30.0	18.0	42.0
Fed		T_max_	minutes	35.4	23.3	30.0	24	54.0

**Table 11:** Comparison of Surge Dose^®^ ibuprofen tablet simulated fasted and fed T_max_ data with published data on ibuprofen lysine tablets.

	T_max_ (minutes)					
Formulation	Arithmetic Mean	CV (%)	Median	Minimum	Maximum	Source
	**Fasted state**					
Ibuprofen lysine salt (Imbun^®^ film tablets)			33.0	24.6	43.2	[27]
Ibuprofen lysine			45.0	30.0	90.0	[28]
Ibuprofen lysine	43.8	41.1				[29]
Ibuprofen lysine	32.52	29.5				[24]
Ibuprofen lysine			30–44			[25]
Surge Dose^®^ ibuprofen	31.2	17.1	30	18	42	Surge Dose^®^ Simulation
	**Fed state**					
Ibuprofen lysine		90	60	180	90	[28]
Ibuprofen lysine salt (Imbun^®^ film tablets)		70.8	29.4	135.6	70.8	[27]
Surge Dose^®^ ibuprofen	35.4	23.3	30	24	54	Surge Dose^®^ Simulation

## Data Availability

The in vitro and in vivo data on Surge Dose^®^ formulations referred to in this paper and used for the modelling are covered by confidentiality and non-disclosure agreements.

## Supplementary Materials

The following supporting information can be downloaded at https://www.mdpi.com/article/10.3390/pharmaceutics17060708/s1: Figure S1: Compartmental PK models schematics; Figure S2: Ibuprofen two-compartment human IV model; Figure S3: PPSA on gastric MTT PK profiles; Figure S4: PPSA on gastric mean precipitation time PK profiles; Equations (S1)–(S9): Differential equations of the compartmental PK models; Equations (S10) and (S11): The one phase and two phases of the Weibull model equations; Equations (S12)–(S17): Two-phase fed-state model and its application to the GastroPlus^®^.
