# Supplementary material for: Surge Dose® Formulations of NSAIDs Provide for Ultra-Rapid and Consistent Drug Absorption in Both the Fasted and Fed State as Predicted by Physiologically Based Biopharmaceutics Modelling
Source: Pharmaceutics. 2025 May 28;17(6):708. doi: 10.3390/pharmaceutics17060708 (PMC12195912; doi:10.3390/pharmaceutics17060708)
Supplement: Supplementary file 1 [file pharmaceutics-17-00708-s001.zip › pharmaceutics-3565484-supplementary.pdf]

Surge Dose® formulations of NSAIDs provide for ultra-rapid and consistent drug absorption in both the fasted and fed state as predicted by Physiologically Based Biopharmaceutics Modelling

Dickinson[1] H, Jiang[1,4] Z, Dickinson[1] PA, Wilding[2] IR and Elliott[3*] GA

Affiliation
[1] Seda Pharmaceutical Development Services, Oakfield Road Cheadle Royal Business Park, Cheadle, SK8 3GX, UK
[2] Ian Wilding Associates Limited, 10 Glebe Street, Nottingham, NG9 1BZ, UK
[3] Imaginot Pty Ltd, Yeerongpilly Corporate Park, 44 Station Street, Yeerongpilly, QLD 4105, Australia
[4] Current address: Department I of Pharmacology, Faculty of Medicine and University Hospital Cologne, University of Cologne, Cologne, Germany
[*]Correspondence: gelliott@imaginot.com.au

Supplementary S1

Compartmental pharmacokinetics (PK) model differential equations and schematics.

Differential equations of the compartmental PK models:

$$\frac{dcentral}{dt} = -\frac{CL}{V} * central \quad \text{Eq. S1}$$

$$\frac{dcentral}{dt} = -\frac{CL}{V} * central - k_{12} * central + k_{21} * peri \quad \text{Eq. S2}$$

$$\frac{dcentral}{dt} = -\frac{CL}{V} * central - k_{12} * central + k_{21} * peri - k_{13} * central + k_{31} * peri2 \quad \text{Eq. S3}$$

$$\frac{dperi}{dt} = k_{12} * central - k_{21} * peri \quad \text{Eq. S4}$$

$$\frac{dperi2}{dt} = k_{13} * central - k_{31} * peri2 \quad \text{Eq. S5}$$

$$central(0) = dose \quad \text{Eq. S6}$$

$$peri(0) = 0 \quad \text{Eq. S7}$$

$$peri2(0) = 0 \quad \text{Eq. S8}$$

$$Concentration = central/V \quad \text{Eq. S9}$$

Where *CL* represents the clearance of the compound; *V* represents the volume of the central compartment; *central* represents the concentration in the central compartment; *peri* and *peri2* represent the amount in the first and the second peripheral compartments; k_{12}, k_{21}, k_{13} and k_{31} represent the distribution constant rates; peri: peripheral.

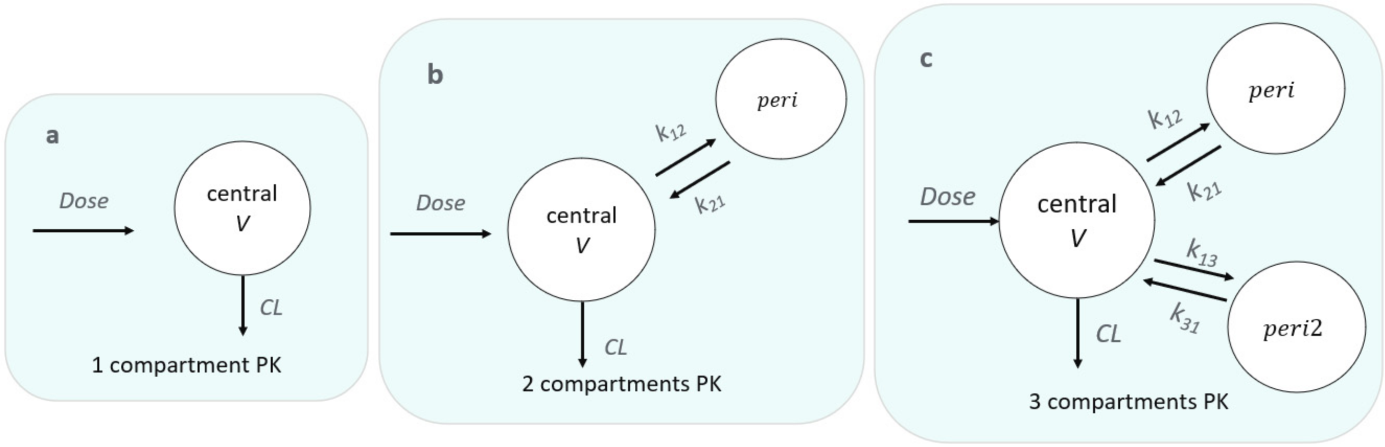

Figure S1. Compartmental PK models schematics. (a) Schematic of one compartment PK model; (b) Schematic of two-compartment PK model; (c) Schematic of three-compartment PK model. CL: the clearance of the compound; V: the volume of the central compartment; k_{12}, k_{21}, k_{13} and k_{31}: the distribution constant rates; peri: peripheral.

Supplementary S2

Weibull function in GastroPlus®

One phase Weibull model:

$$f_t = f_{max}\left(1 - e^{-\left(\frac{t-t_{lag}}{\alpha}\right)^{\beta}}\right) \quad \text{Eq. S10}$$

Two phases: Weibull model:

$$f_t = f_{max}\left(1 - e^{-\left(\frac{t-t_{lag}}{\alpha 1}\right)^{\beta 1}} - e^{-\left(\frac{t-t_{lag}}{\alpha 2}\right)^{\beta 2}}\right) \quad \text{Eq. S11}$$

Supplementary S3

Two-phase fed state model and its application to the GastroPlus®

Equations of the early phase of the fed state model:

$$\frac{dG_t}{dt} = K_{GE(fed)3} * \left(X_t - X_{1.5h}\right) \quad \text{Eq. S12}$$

$$K_{GE(fed)3} = 7.21 \quad \text{Eq. S13}$$

Equations of the late phase of the fed state model:

$$\frac{dG_t}{dt} = K_{GE(fed)4} * X_t \quad \text{Eq. S14}$$

$$K_{GE(fed)4} = 0.452 \quad \text{Eq. S15}$$

Where G_t and X_t represent the drug amount that has been emptied from the stomach or the drug amount that remains in the stomach, respectively, at a given time t. $K_{GE(fed)}$ represents the gastric emptying constant rate, expressed in units of /h.

To investigate the correlation between the gastric emptying constant rate ($K_{GE\ (fed)}$) and the gastric *MTT* (in units of hours) as the GastroPlus® input, a nominal gastric MTT of 2 hours was set in GastroPlus® and the resulting gastric emptying curve was extracted. The resulting profile was compared to profiles generated using the ordinary differential equations (ODE) Eq. S16 – Eq. S17.

$$\frac{dX_t}{dt} = -K_{GE(fed)} * X_t \qquad \text{Eq. S16}$$

$$X_t(0) = 50 \qquad \text{Eq. S17}$$

Supplementary S4

Fitted two-compartment model to the ibuprofen IV data

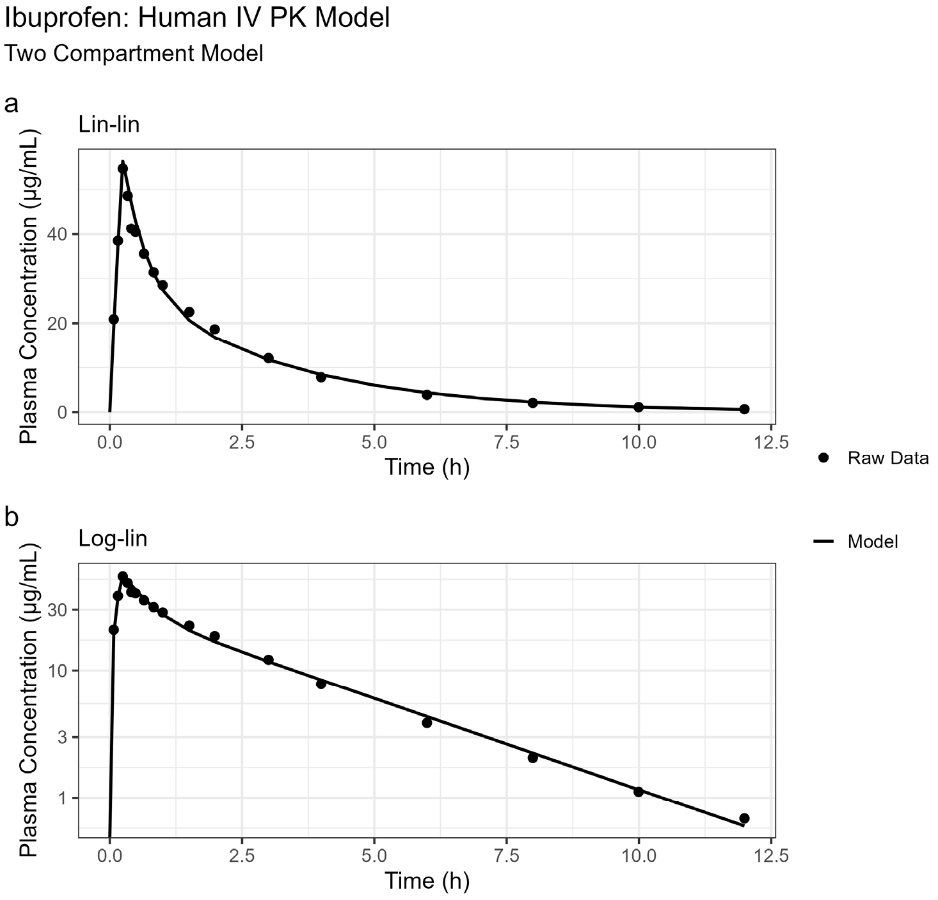

Figure S2. Ibuprofen two-compartment human IV model. The line is the simulated model, and the points are the sum of the raw r-ibuprofen and s-ibuprofen data at each time point. (a) Plot is on a linear-linear scale. (b) Plot is on a log-linear scale.

Supplementary S5

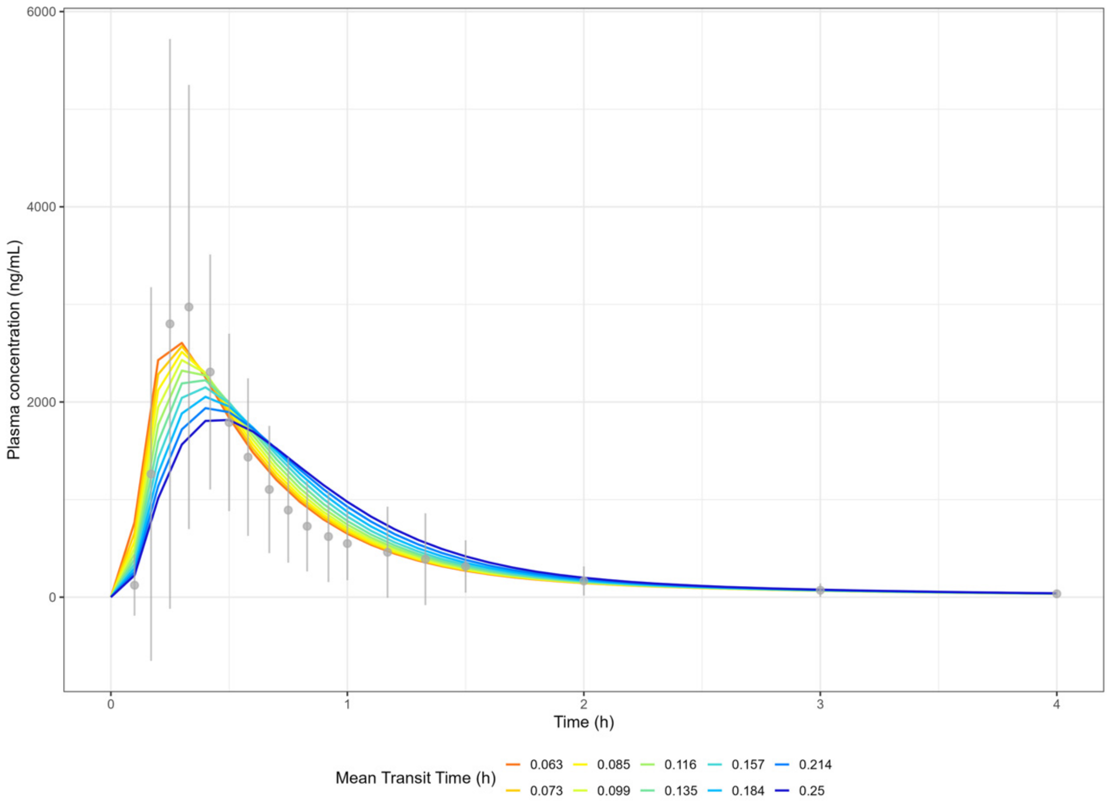

Figure S3. PPSA analysis showing the impact of changes in the gastric MTT on the resulting PK for oral diclofenac Surge Dose® tablets in the fasted state.

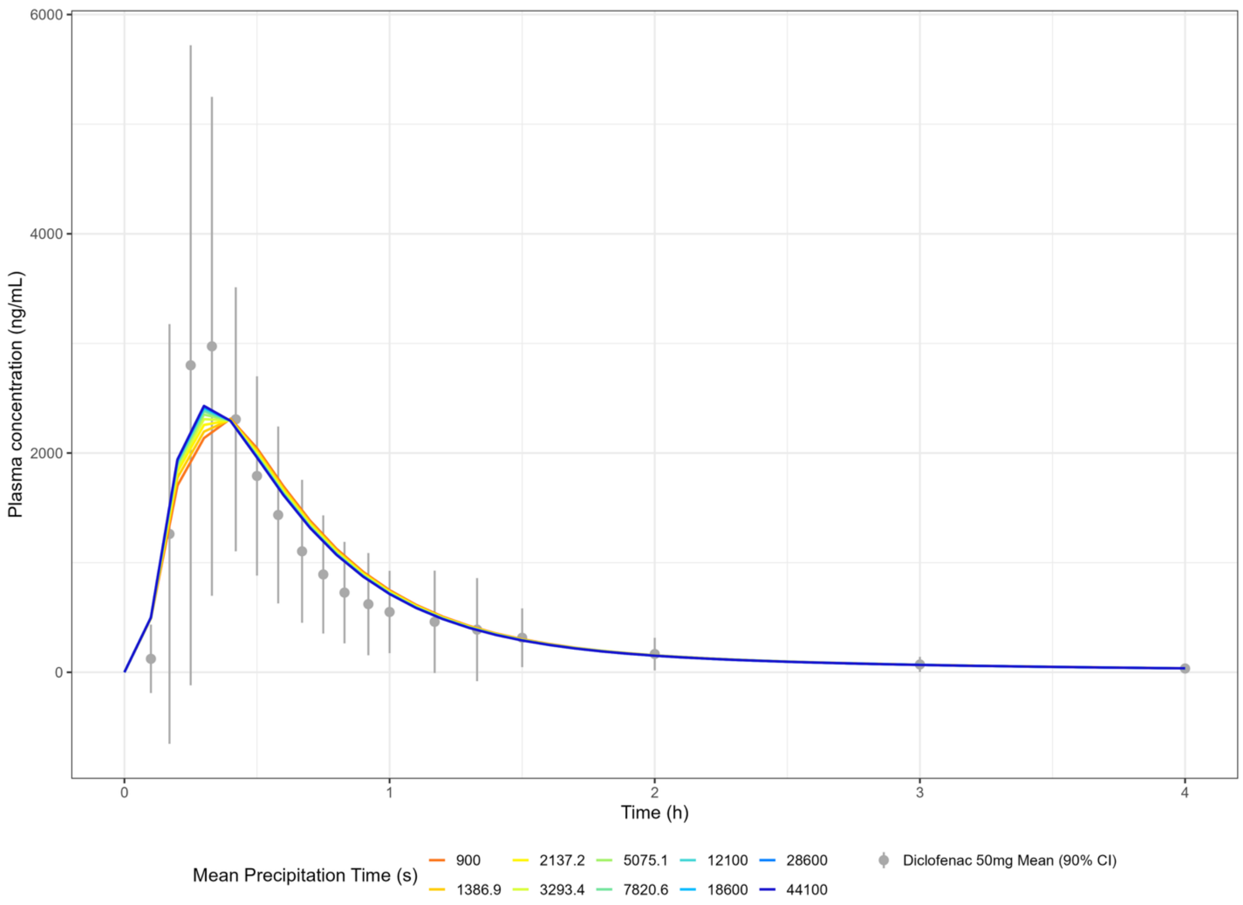

Figure S4. PPSA analysis showing the impact of changes in the mean precipitation time on the resulting PK for oral diclofenac Surge Dose® tablets in the fasted state.
